# Conformationally Restricted Peptides from Rice Proteins Elicit Antibodies That Recognize the Corresponding Native Protein in ELISA Assays

**DOI:** 10.3390/molecules23092262

**Published:** 2018-09-05

**Authors:** Nubia F. Barrera, Luz M. Melgarejo, Maribel Cruz-Gallego, Lina Jimena Cortés, Fanny Guzmán, Julio C. Calvo

**Affiliations:** 1 Doctorado en Biotecnología, Universidad Nacional de Colombia, Carrera 30 No. 45-03, Edificio 224, Bogotá 110111, Colombia; nfbarrerac@unal.edu.co; 2 Grupo Proteoma UD, Facultad de Ciencias y Educación, Universidad Distrital Francisco José de Caldas, Carrera 4 No. 26B-54, Bogotá 110111, Colombia; 3 Laboratorio de Fisiología y Bioquímica Vegetal, Departamento de Biología, Universidad Nacional de Colombia, Carrera 30 No. 45-03, Edificio 421, Bogotá 110111, Colombia; lmmelgarejom@unal.edu.co; 4 Centro Internacional de Agricultura Tropical, CIAT, Fondo Latinoamericano para Arroz de Riego, FLAR, Palmira, Valle 763537, Colombia; maribel.cruz@cgiar.org; 5 Núcleo de Biotecnología de Curauma, Pontificia Universidad Católica de Valparaíso, Valparaíso 2373223, Chile; lina.cortes@pucv.cl (L.J.C.); fanny.guzman@pucv.cl (F.G.)

**Keywords:** plant proteomics, RHBV, rice hoja blanca virus, Colombian rice varieties, peptide dendrimers, helix mimetics

## Abstract

The rice hoja blanca virus (RHBV), transmitted by the planthopper insect *Tagosodes orizicolus*, is a disease that attacks rice and generates significant production losses in Colombia. Fedearroz 2000 and Colombia I commercial rice varieties, which have different resistance levels to the disease, were selected in this study. To identify proteins associated to the insect and virus signaling, a comparative proteomics study was performed. By comparing proteomic profiles, between virus-infected and control group plants in two-dimensional electrophoresis, proteins exhibiting significant changes in abundance were found. In another test, peptide dendrimers containing sequences conformationally restricted to α-helix from four of those rice proteins were synthesized. In the experiment, sera from mice inoculated with peptide dendrimers could recognize the corresponding native protein in ELISA assays. Reported comparative proteomic results provide new insights into the molecular mechanisms of plant response to the RHBV and comprehensive tools for the analysis of new crop varieties. Besides, results from conformational peptide dendrimer approach are promising and show that it is feasible to detect proteins as markers, and may have biological applications by decreasing the susceptibility to proteolytic degradation.

## 1. Introduction

Rice (*Oryza sativa* L.) has become the most important food grain in Latin America and the Caribbean. Rice is also considered a model species in molecular genetics because of the relatively small size of its genome. The rice genome consists of about 466 million base pairs and about 46,000 to 55,000 genes [1]. However, due to post-transcriptional and post-translational modifications, it is not always possible to predict the exact functions of the genes that are involved in different responses to multiple conditions [2,3], including biotic and abiotic stress [4,5]. Similarly, the study of the rice genome has revealed extensive regions of preserved and ordered gene content, whose data can be used to perform various studies for improving this crop [1]. 

Rice hoja blanca (RHB) disease has been reported in tropical and subtropical American countries that grow rice. Unlike the phytosanitary situation of rice in Asia, RHBV was the only viral disease in America until 1991 when the rice stripe necrosis virus emerged in South America [6]. RHBV is transmitted by the planthopper insect *Tagosodes orizicolus,* which grows up to an average size of 2.5 mm. *T. orizicolus* requires high temperatures (about 27 °C) and humidity (>80% RH), and is also a direct rice pest [6,7]. Epidemics of RHB occur sporadically, but with catastrophic results in terms of rice crop yields [8]. Distribution of RHBV is determined by environmental conditions that favor the reproduction and survival of its vector. A potential planthopper vector requires up to 12 h to acquire the virus from an infected rice plant, and a minimum of a week to a month to complete the incubation inside the hopper insect *T. orizicolus* [6]. Also, the virus could be transmitted in a transovarial way; it means from the mother to the progeny. The symptom in the rice plants is the appearance of chlorotic streaks that can coalesce and cause the leaves to turn yellow or white. When young plants become infected, they are stunted, and in severe infections the leaves turn necrotic and the plants die. Infections that occur before the emergence of the panicle can reduce seed set and grain quality. There is indirect evidence that rice infected by RHBV may be susceptible to other diseases. The RHBV virus was isolated and partially characterized as a member of the Tenuivirus genus by Morales and Niessen [7]. The level of resistance of a genotype to rice hoja blanca virus (RHBV) is determined by the percentage of plants that become infected. A screening for RHBV relies on a complex biological system that involves vector colonies, the concentration of the virus in the vector, and environmental conditions that can affect the feeding behavior of the vector insects. In recent years, many scientists have focused on studies of proteins that occur under various conditions in a proteomic analysis. Proteomic techniques have been used to analyze expression patterns of complex mixtures and to explore gene functions in different tissues and the expression under stress, for which the genetic factors involved are identified or associated with defense mechanisms in plants [9,10]. There is a need in Colombia to increase the number of resistant varieties to various stresses caused by climate change effects or diseases. In that direction, a set of virus-responsive proteins was established, and a new approach based on conformationally restricted peptide dendrimers was proposed to detect those proteins [11].

In the present study, Fedearroz 2000 and Colombia 1 rice varieties were evaluated. The Colombia 1 variety was used in all commercial crossings with resistance to RHBV, and has been used as a resistant control assessment at the Centro Internacional de Agricultura Tropical (CIAT) until the appearance of Fedearroz 2000 variety. This variety is resistant to diseases and has higher productivity than other varieties used in Colombia. The first comparative proteomics study between both varieties for evaluating the differential response of rice against the vector and the virus is reported in this study. By performing two-dimensional electrophoresis (2-DE) and MALDI-TOF/TOF mass spectrometry analysis of rice leaves, a set of vector- and virus-responsive proteins was established. Virus-infected plants showed significant differences in morphology and metabolism, compared to normal plants (control group). In another test, we identified some of these low concentrated proteins in leaf extracts using peptide dendrimers by immunoenzymatic methods with good results. To get these results, a new approach based on design and synthesis of conformationally restricted peptide dendrimers was proposed. Proteomic results provide new insights into the molecular mechanisms of plant response to virus infection and comprehensive tools for the analysis of new crop varieties. ELISA results are promising and show that it is feasible to design and synthesize peptide dendrimers with target sequences conformationally restricted to α-helix to be used for biological applications.

## 2. Results

Plants were cultivated in a growth chamber under 12/12 h light/dark cycle at a temperature of 25 °C, and relative humidity of 77%. The plants were irrigated daily with water for approximately 30 days to keep the soil moist. The unstressed and stressed plants were kept in the same growth chamber.

The evaluation of the results was made based on the analysis of the three treatments: (1) control group; (2) vector without RHBV; and (3) vector infected with RHBV, and compared for the two rice varieties used, Fedearroz 2000 and Colombia I, as indicated in the subsequent sections.

### 2.1. Analysis of Viral Presence

**Figure 1:**
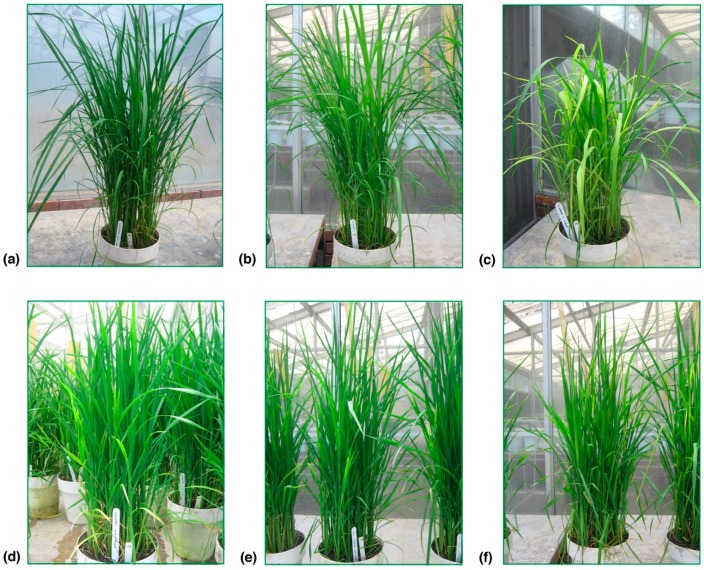
The effect of the insect and RHBV exposure of the two varieties. Colombia I variety: control (**a**), non-infected insect (**b**), and insect infected with RHBV (**c**). Fedearroz 2000 variety: control (**d**), non-infected insect (**e**), and insect infected with RHBV (**f**).

In the present study, Fedearroz 2000 and Colombia 1 rice varieties were evaluated. Colombia 1 variety was used in all commercial crossings with resistance to RHBV. It is an old variety that carried this resistance but has lost it over time. Colombia 1 variety was used as a resistant control assessment until the appearance of Fedearroz 2000 variety, which is a highly virus-resistant commercial rice variety with increased productivity with respect to other varieties used in Colombia. These materials have been used as a control in developed assessments by the International Center of Tropical Agriculture (Centro Internacional de Agricultura Tropical; CIAT, Palmira, Valle, Colombia) and Latin American Fund for Irrigation Rice (Fondo Latinoamericano para Arroz de Riego; FLAR; Palmira, Valle, Colombia). Thirty days after infection, symptoms of the disease were corroborated. Symptoms such as light-colored bands on the leaves (chlorosis), stunted leaves and wilting in plants exposed to the insects that carry the RHBV were observed in Colombia I rice variety (Figure 1a–c) and in Fedearroz 2000 variety (Figure 1d–f). The percentage of plants showing symptoms of the disease in the RHBV infection treatment were 50% for Fedearroz 2000 variety and 85.5% for Colombia I variety. Control and insect without RHBV groups showed no symptoms of the disease. The presence/absence of the virus was confirmed by ELISA using the rabbit antiserum against the coat protein (CP) of RHBV. Results indicated no contamination between treatments.

### 2.2. Comparative Analysis of 2-DE

**Figure 2:**
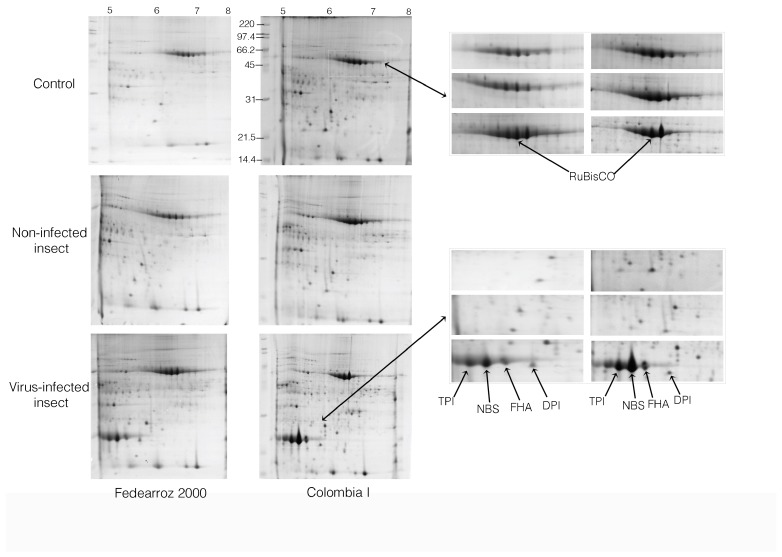
Gels obtained in the experiment contains spots for each treatment of the Fedearroz 2000 virus resistant commercial variety and Colombia I variety. Right panel shows a zoom of the regions indicated in the gels, and the organization follows the same pattern as the left panel. Images were created using the BioRad PDQUEST program, version 8.0.1. The spots indicated are RuBisCO: Ribulose-1,5-bisphosphate carboxylase/oxygenase; DPI: Adenosine diphosphate glucose pyrophosphatase; TPI: Triosephosphate isomerase; NBS: NBS-containing resistance-like protein containing protein; FHA: FHA domain containing protein DDL. Molecular weight (*y*-axis) and pH (*x*-axis) are indicated.

Approximately 4000 spots were stained with Coomassie blue G-250 and were reproducibly resolved in all 2-DE gels in the comparison analysis. The gels were digitized and normalized using the PDQuest program (BioRad, version 8.0.1, Hercules, CA, USA). Gels for each treatment and for each variety are showed in Figure 2. The statistical analysis was carried out with the software SPSS v.10.0. The differential variations in the abundances of the spots were tested with ANOVA test (α = 0.05) for each treatment. Inconsistent spots were not taken into account and removed from the database. The averages of significant spots were compared with the Student’s *t*-test. For protein identification, a combined search was performed with MASCOT search engine over NCBInr Viridiplantae.

Analysis of gel images matched 374 reproducibly spots for the three treatment replicas and two varieties. In this case 95 spots (26%) were found in common in the two varieties under study, 136 spots (36%) only in the Fedearroz 2000 variety and 143 spots (38%) only in the Colombia I variety.

**Figure 3:**
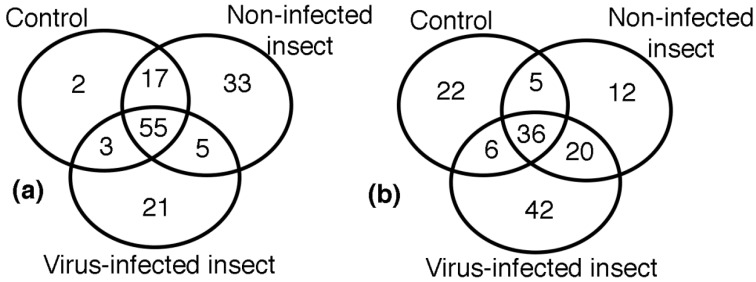
Venn diagram representing differentially expressed spots for the three experimental groups: treatment 1 (control group), treatment 2 (non-infected insect), and treatment 3 (insect infected with RHBV). (**a**) Fedearroz 2000 variety; (**b**) Colombia I variety.

Plant resistance to pathogens involves a series of inducible defensive responses which include the synthesis of various pathogenesis-related proteins and multiple isoenzymes with antioxidant activity. The inducible defensive response of plants is activated when they are infected by pathogens. Analysis of images in the virus resistant commercial variety revealed the distribution of 136 reproducibly spots in the three treatments, as it is shown in the Venn diagram of Figure 3a. The analysis of images of the three treatments in the Colombia I variety (143 spots) is shown in Figure 3b.

Common proteins identified in the two varieties under study are listed in Table 1. 

The column ‘Treatment: Insect’ compares differentially-expressed proteins under non-infected insect treatment, between Fedearroz 2000 (Fed.) and Colombia I (Col. I) varieties. In the photosynthesis group of proteins, the first four proteins showed differential expression in the Colombia I variety, while Fedearroz 2000 variety did not show any reaction. In general, the trend was a decrease with respect to the control group. In the antioxidant system group, the trend in Ferredoxin-NADP(H) oxidoreductase and superoxide dismutase proteins were to increase in Fedearroz 2000 variety, and decrease in the Colombia variety. The metabolism group showed opposite trend for the two varieties. In the defense group, the tendency to increase in the Germin-like protein against the attack of the insect is interesting. In the biosynthesis group, the trend was up in Colombia I variety and down in Fedearroz variety. The trend in FHA protein was to decrease in the two varieties.

The ‘Treatment: Virus’ column (Table 1) compares differentially-expressed proteins under RHB virus-infected insect treatment, between Fedearroz 2000 (Fed.) and Colombia I (Col. I) varieties. In the photosynthesis group, proteins presented the same trend. In the antioxidant system group, the trend of the manganese superoxide dismutase protein was to decrease in Fedearroz 2000 variety, and to increase in Colombia I variety. In the metabolism group, the trend of the triosephosphate isomerase was to decrease in Fedearroz 2000 variety, and to increase in Colombia I variety. 

Proteins identified only in Fedearroz 2000 variety are listed in Table 2. Proteins identified only in Colombia I variety are listed in Table 3. The information provided here includes the NCBI accession number, protein score, peptide count, experimental/theoretical molecular masses, isoelectric point, and trend (up/down).

In this work, statistical analysis of gel image revealed that most of the proteins increased in abundance, but some of the protein spots were completely absent in any of the two treatments. From the 95 spots found in the two varieties, 57 relevant spots were selected to identify proteins. From these spots, 34 proteins were identified, because many of the spots corresponded to the same protein. Proteins found were grouped into categories based upon their biochemical functions, including photosynthesis (26.5%), antioxidant system (14.7%), metabolism (17.6%), defense response (8.8%), biosynthesis (2.9%), cellular signaling (5.9%), and unknown functions (23.5%) (Table 1). Statistical analysis of images detected 136 spots found only in the Fedearroz 2000 virus resistant commercial variety. These spots were grouped as follows: 55 spots in common in the three treatments, 2 spots in control group, 33 spots in the non-infected insect group, 21 spots in the virus-infected group, 17 spots in common between control and non-infected insect groups, 5 spots in common between non-infected and virus-infected insect groups, and 3 spots between control and virus-infected groups (Table 2). Statistical analysis of images detected 143 spots found only in the Colombia I variety. These spots were grouped as follows: 36 spots in common in the three treatments, 22 spots in the control group, 12 spots in the insect group, 42 spots in the virus group, 5 spots in common between control and insect groups, 20 spots in common between insect and virus groups, and 6 spots between control and virus groups (Table 3).

### 2.3. Synthesis and Characterization of Peptides

Amphipathic and exposed α-helical fragments on the surface of two common proteins (FHA and ferredoxin-NADP(H) oxidoreductase) and two proteins identified only in Colombia I variety (α-galactosidase and tapetum-specific zinc-finger protein 1) were selected to design and synthesize peptide dendrimers with branches conformationally restricted to α-helix. Helix 1, from FHA protein, is an α-helical structure in the segment encompassing residues 19-29 (LIHKEIKKRTK). Helix 2, from rice α-galactosidase protein, is an α-helical structure in the segment encompassing residues 195–205 (MERYTRMSNAM). Helix 3, residues 32–41 (EDILRSMIKE), and Helix 4, residues 190–198 (QALGGHKRS), are α-helical structures from ferredoxin-NADP(H) oxidoreductase and tapetum-specific zinc-finger protein 1 respectively. These α-helical fragments were selected to design and synthesize peptide dendrimers with branches conformationally restricted to α-helix. A list of synthesized peptides is given in Table 4.

**Figure 4:**
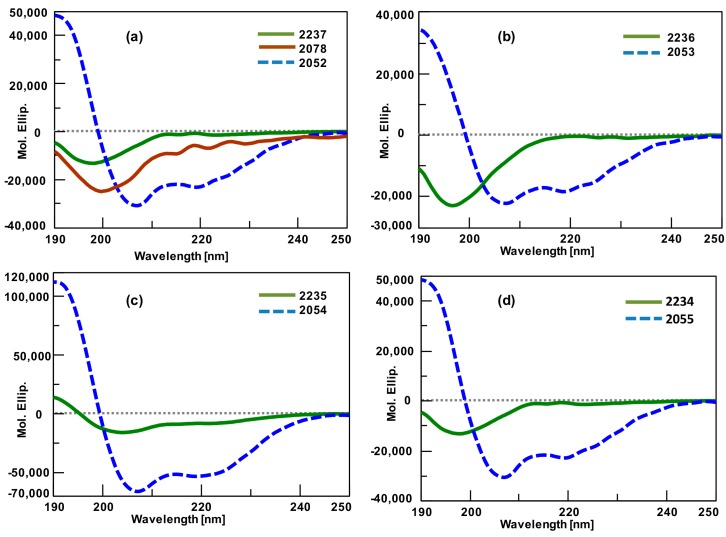
Circular dichroism of linear and conformationally restricted peptides: (**a**) Linear peptides 1 (2237 and 2078), and Helix 1 (2052); (**b**) Linear peptide 2 (2236) and Helix 2 (2053); (**c**) Linear peptide 3 (2235) and Helix 3 (2054); (**d**) Linear peptide 4 (2234) and Helix 4 (2055).

Linear peptides were analyzed by analytical RP-HPLC, and a single major peak was obtained. Peptides had the correct molecular weight as measured by MALDI-TOF mass spectrometry. Based on these results, we proceeded with the insertion of the JAK capping sequence and cyclization to obtain the peptides conformationally restricted to α-helix. Linear and conformationally restricted peptides were analyzed by CD. Their CD spectra are shown in Figure 4. CDPro software package was used for analyzing the peptide dendrimers CD spectra in order to determine the secondary structure fractions. After CD analysis, dimers were oxidized (10 mg/mL) in 10% DMSO at pH 7.0 and tetramer (double dimer constructs, DDCs) formation was monitored following a previously described procedure [12].

### 2.4. Biological Assays

Once it was confirmed that it was possible to synthesize peptide dendrimers with branches conformationally restricted to α-helix, peptides were inoculated in Balb-C mice, to verify that they were immunogenic and able to induce the appropriated immune response. When assessed, the antibody titers against the corresponding peptides were ≥1:25,600 after the third dose.

Leaf extracts from plants of the control group, from plants exposed to the insect without virus (Insect Group), and plants exposed to the insect infected with RHBV (Virus Group) were used for the specific serological recognition of proteins (Table 5). Sera were collected from mice inoculated with peptide dendrimers containing sequences derived from rice proteins.

Among the 46 response proteins to insect and RHBV being identified, the FHA protein, rice α-galactosidase protein, ferredoxin-NADP(H) oxidoreductase protein, and tapetum-specific zinc-finger protein, give an overview of the differential response in the two rice varieties. For these low-concentrated proteins, the cutoff value represents an absorbance two times greater than that of the blank. Sera with absorbance values greater than the cutoff were considered positive.

## 3. Discussion

The study was designed to detect differences in protein expression between two rice Colombian varieties under exposure with RHBV and its vector (*Tagosodes orizicolus*). The presence of the insect generated mechanical damage by the feed form and oviposition of the vector. According to the results obtained in the infectivity assay, the described varieties may suffer the disease, with the Colombia I variety being the most affected. Sixteen-day-old plants were placed in the presence of the vector and virus and four insects per plant. These conditions are stronger than those found in the field, and that is probably why the symptoms in the Fedearroz 2000 virus resistant commercial variety could be observed [13,14]. Nucleotide binding sites (NBS) containing resistance like protein, RuBisCo and triose phosphate isomerase were identified in more than one spot probably due to the presence of different protein isoforms, post-transductional modification or degradation [15]. Together, these results suggest changes in the biological processes of adaptation into Fedearroz 2000 and Colombia I rice varieties. Plant resistance to pathogens involves a series of inducible defensive responses, which include the synthesis of various pathogenesis-related proteins and multiple isoenzymes with antioxidant activity. Our results showed that expression of several proteins increased in severely infected plants compared to normal plants. These proteins contain characteristic domains of a large family of plant R genes, including leucine-rich repeats (LRR) domain, nucleotide-binding sites (NBS) domain, and/or toll/interleukin-receptor similarity (TIR) domain. Upregulation of these proteins in virus infected plants with RHBV suggests that those proteins may be involved in the defensive response to RHBV.

Compared to those in normal plants, fructose bisphosphate aldolase, adenosine diphosphate glucose pyrophosphatase, NAD-dependent putative epimerase/dehydratase, and GADPH (glyceraldehyde-3-phosphate dehydrogenase) showed variations between virus-infected and normal plants, which suggests that the capability of carbon fixation and assimilation might be relatively enhanced in virus-infected plants, particularly in the Colombia I variety. Glutamine synthase, which is an important enzyme in the ammonium assimilation process, was detected in all treatments for Fedearroz 2000 variety and in the insect and virus treatment for Colombia I variety. Differential accumulation of these proteins implies a shift of sugar metabolism after RHBV occurrence, which is likely to be an important reason for drastic morphological and physiological modifications caused by RHBV infection. The relative abundance of proteins involved in amino acid metabolism showed differences between virus-infected and control group plants. These results suggest that the effects of RHBV infection were also reflected in a dynamic modification of both amino acid catabolism and anabolism, and also suggest the specificity for synthesis and/or degradation of amino acids after RHBV occurrence. The expression changes of proteins related to carbon metabolism indicate that the fundamental metabolism in virus-infected plants with RHBV disease is seriously impaired, which ultimately results in significant differences in morphology and development between virus-infected and normal plants.

From the 136 spots found only in the Fedearroz 2000 virus resistant commercial variety, 22 relevant spots were selected and 9 proteins were identified by mass spectrometry. Proteins were grouped as biosynthesis-related proteins, metabolism-related proteins, and proteins of unknown function (Table 2). These data suggest that 60% of the spots identified may correspond to post-translationally modified forms, members of multigene families, to products of protein degradation or to products of alternative splicing [16,17]. Transketolase 1, a metabolism-related protein, increased in insect-treated plants; it is related to the Calvin cycle and the pentose phosphate pathway. Similar results were found by Manaa et al. under conditions of oxidative stress and high salinity in corn and tomato plants [18]. The importance of this enzyme is that, in the pentose phosphate pathway, it produces NADPH needed for different pickers reactive oxygen species (ROS) systems. Phosphoglycerate kinase was identified as an Upregulated protein only in the virus group. Previous reports showed that proteins involved in carbohydrate metabolism were up/downregulated under selenium treatment [19]. We found proteins involved in carbohydrate metabolism up/downregulated, such as chloroplast phosphoglycerate kinase, which is upregulated under virus-infected insect treatment in the Fedearroz 2000 variety. We also found that the membrane anchored protein ubiquitin fold protein 4, detected in the control group, was downregulated under non-infected insect and virus-infected insect stress.

From the 143 spots found only in the Colombia I variety, 78 relevant spots were selected and 22 proteins were identified by mass spectrometry. Proteins were grouped into five categories: photosynthesis-related, metabolism-related, plant defense-related, biosynthesis-related, and signaling and intracellular transport-related (Table 3). Photosynthesis-related proteins. LHCII type I chlorophyll a/b binding protein showed variations in its intensity. Previous reports showed that this protein increased in rice plants treated with selenium [19]. It is likely that accumulated pigments can promote photochemical reactions in the chloroplast to produce reducing equivalents (ATP and NADPH) for carbon fixation; it indicates that the plant is possibly recovering from the insect feeding process and/or responses against viruses [19]. This is equivalent to say that these proteins may be important in the recovery process of the plant. Asada [20] reported that proteins that prevent damage by ROS are important in the chloroplast to protect the photosynthetic apparatus.

Metabolism-related proteins. The Rieske iron–sulfur protein family showed a decrease in abundance under insect and virus stress. Some of the proteins found in the present study have been reported by Brizard et al. [21], who studied the interaction of the yellow mottle virus in rice. On the other hand, the production of ROS in stress can be increased by photosynthesis, respiration, and NADPH oxidation processes, and as a result of the rupture of photosystems [22], which is why most of the amount of Rubisco subunits identified in this work may be related to the stress generated by the virus in plants of the Colombia I variety.

Once the identification of differentially expressed proteins was completed, we wanted to know if it was possible to identify these low concentrated proteins in leaf extracts by immunoenzymatic methods. Amphipathic and exposed α-helical fragments on the surface of two common proteins (FHA protein and ferredoxin-NADP(H)oxidoreductase) and two proteins identified only in Colombia I variety (rice α-galactosidase and tapetum -specific zinc-finger protein 1) were selected. Because of their flexible nature, short peptides often exhibit reduced target affinity and low proteolytic stability. Peptide dendrimers have usually higher activity than monomers, because of their higher concentration of bioactive units, as well as their higher proteolytic stability. The DDCs method was chosen to obtain peptide dendrimers, because it was demonstrated that DDCs can be obtained rather easily as chemically defined molecules [12,23].

The approach was intended for stabilizing peptides in α-helix conformation using the JAK nucleation site in peptide dendrimers. Peptide dendrimers containing the helix 1, helix 2, helix 3, and helix 4 sequences were synthesized and products without the JAK capping sequence had the correct molecular weight (Table 4). Then, the insertion and cyclization of the JAK capping sequence were carried out for obtaining the α-helical mimics. Peptide dendrimers with the JAK nucleation site produced CD spectra that were typical of α-helix, while peptides without the JAK nucleation site showed spectra with no helical conformation (Figure 4). The helical content of peptide dendrimers was strongly increased by the insertion of the JAK nucleation site. Results showed that the ability of the JAK nucleation site to mimic the α-helical structure was derived primarily from the degree of helicity of the target sequence. 

Other approaches to helical mimetics considered the modifications of sidechains, or used unnatural amino acids [24,25]. These modifications of sidechains might modify one face of the helix or might require the replacement of key recognition sidechains. Another approach, using a hydrazone link nucleation site in short peptides, showed good results in the identification of conformationally sensitive antibodies in human sera [26]. Unfortunately, the insertion of the hydrazone link requires the prior preparation of two expensive linkers [27]. In addition, the hydrazone link nucleation site is acid sensitive and it is not easy to insert in multimeric molecules. The JAK nucleation site approach uses the target sequences without modifications and it can be used in multimeric molecules. Linker J (propanedioic acid) is a common and low-cost reagent, and linker K (Fmoc-Lys(Dde)-OH) is a protected amino acid used in peptide synthesis. 

Assays for specific serological recognition of proteins by using sera from mice, inoculated with peptide dendrimers with branches conformationally restricted to α-helix, were relatively successful. Sera from mice inoculated with peptide dendrimer 2052 (FHA protein) recognized the protein extracts of the Fedearroz 2000 and Colombia I varieties in all treatments. This protein was observed to have an uptrend in all the treatments for the two varieties, except for insect treatment in Colombia I. Sera from mice inoculated with peptide dendrimer 2054 (ferredoxin-NADP(H) oxidoreductase protein) recognized leaf extracts from Fedearroz 2000 in control and insect treatments and only recognized leaf extracts in control group from Colombia I variety. As can be seen in Table 1, this protein, even though expressed in the two varieties, has a downtrend in insect and virus treatment for Colombia I variety. These proteins were identified as common and differentially expressed proteins in both varieties. Sera from mice inoculated with peptide dendrimer 2055 (tapetum-specific zinc-finger protein) recognized leaf extracts from Colombia I variety in control and virus treatments and showed no reaction with leaf extracts from Fedearroz 2000 variety. Sera from mice inoculated with peptide dendrimer 2053 (α-galactosidase) only recognized the control leaf extract from Colombia I variety. The response for the proteins from Colombia I variety was detected in the cases it showing an uptrend, as can be seen in Table 3. 

With the serological assays, it was possible to identify two of the common proteins and two from the Colombia variety, showing similarity to those from proteomics, with the exception of the FAH protein in insect treatment for Fedearroz variety, which could be a concentration effect in the ELISA assay detection, which is a semiquantitative technique.

## 4. Materials and Methods

### 4.1. Plant Growth and Infectivity Assays

Fedearroz 2000 is a highly virus resistant commercial rice variety, and Colombia I is an old variety that carried this resistance but has lost it over time. These materials have been used as control in developed assessments by the Centro Internacional de Agricultura Tropical (CIAT) and Fondo Latinoamericano para Arroz de Riego (FLAR). The experiments were conducted using a randomized complete block design with three biological replicates; each replica consisted of 40 plants, for a total of 120 plants per group, two varieties and three experimental conditions. Seeds were sown in plastic pots (20 cm in diameter and 15 cm in high) filled with soil. Plants were raised in a growth chamber under a 12/12 h light/dark cycle at a temperature of 25 °C, and relative humidity of 77%. The plants were irrigated daily with water for approximately 30 days to keep the soil moist. The unstressed and stressed plants were kept in the same growth chamber.

On the 16th day, plants at the two-leaf stage were treated for infectivity assays: one-third of plants were used as a control group, one-third were exposed to the vector without RHBV (four planthoppers per plant for three days) according to Morales & Jennings [6], and one-third were exposed to the vector infected with RHBV (four planthoppers per plant for three days); finally, insects were removed with water. Thirty days after infection, symptoms of the disease were observed and the percentage of plants in each variety showing symptoms of the disease was determined. An ELISA test was performed to confirm the presence/absence of the virus. Extracts of rice leaves of exposed and non-exposed plants to the virus, and a rabbit antiserum against the coat protein (CP) of RHBV were used for the antigen-antibody reaction [8].

The leaf extracts of the three treatment plants and varieties were prepared by macerating 1 g of the plant material in 0.1 M PBS buffer, pH 7.4/Tween 20 0.05%, pressing the macerated material, and adding PBS buffer to obtain 5 mL. ELISA plates were coated with the rabbit antiserum against the coat protein (CP) of RHBV diluted 1/4000 in 0.05 M sodium carbonate buffer, pH 9.6. After an incubation period of 5 h at 37 °C, plates were washed three times with 0.1 M PBS buffer, pH 7.4/Tween 20 0.05%, 200 µL/well of leaf extracts were added, and the plates were incubated overnight at 4 °C. Plates were washed three times with 0.1 M PBS buffer, pH 7.4/Tween 20 0.05%, 50 µL/well of alkaline phosphatase-labeled IgG conjugate was added, and the plates were incubated for 5 h at 37 °C. Plates were washed three times with 0.1 M PBS buffer, pH 7.4/Tween 20 0.05%, and substrate was added to each well at a concentration of 1 mg/mL in 10% diethanolamine buffer, pH 9.8, containing 0.02 sodium azide. After a period of 15 min, the optical densities were determined at 405 nm wavelength, using a Dynex MRX ELISA reader (Markham, ON, Canada)

### 4.2. Protein Extraction 

Proteins from leaf tissues of the three treatments were extracted by the trichloroacetic acid (TCA)/acetone method, as previously described with some modifications [28]. In brief, about 0.5 g of frozen samples were ground in liquid nitrogen to a fine powder and incubated overnight in 10 volumes of ice-cold 10% (*w*/*v*) TCA in acetone with 0.5% (*w*/*v*) DTT (dithiothreitol) at 20 °C, and then centrifuged at 15,000 g for 15 min at 4 °C. The supernatants were discarded and the pellets were washed three times with 8 mL of ice-cold acetone containing 0.5% DTT, and then dried overnight. Protein concentration was measured according to a modified Bradford assay using a Sigma kit and bovine serum albumin (1.5 µg/µL) as standard.

### 4.3. Two-Dimensional Electrophoresis (2-DE) and Image Analysis

Separation by 2-DE, image analysis and mass spectrometry was performed as previously described [29]. For each treatment, three biological replicates of 2-DE were conducted using different plant materials. For Isoelectric focusing (IEF), proteins were dissolved in a buffer containing 9 M urea, 4% (*w*/*v*) 3-[(3-cholamidopropyl) dimethylammonio]-1-propanesulfonate (CHAPS), 0.5% (*v*/*v*) Triton X-100, 20 mM DTT, 1.2 % (*v*/*v*) pharmalytes pH 3–10, and kept for two hours at 4 °C. Then, 400 μg of protein samples were loaded onto IPG strip holder. Immobilized linear gradient strips (pH 5–8, 18 cm, Bio-Rad, Hercules, CA, USA) were rehydrated for 14 h at 20 °C. IEF was performed using a Protean cell system (Bio-Rad) at 20 °C by ramping to 500 V for 1 h, holding at 500 V for 1 h, and 1000 V for 1 h successively, ramping to 8000 V for 1 h, and holding at 8000 V, until reaching a total of 32 kVh. Prior to the second-dimension separation, the gel strips were equilibrated in equilibration buffer (6 M urea, 30% (*w*/*v*) glycerol, 2% (*w*/*v*) SDS, 50 mM Tris–HCl, pH 8.0), first with 1% DTT and then with 2.5% iodoacetamide, each for 15 min. The strips were then transferred to 12.5% vertical SDS-PAGE gels for the second dimension electrophoresis using a Dodeca Vertical System (Bio Rad). SDS-PAGE was run at 3 W/gel for 12–16 h until the bromophenol blue dye front reached the gel end. Gels were stained with CBB G-250 and then the stained gels were scanned using ImageScanner (Bio Rad) at a 300 dpi resolution. Gel images were analyzed using the PDQuest 2D Analysis software v. 7.0 (Bio Rad). When comparing three or more gels, the evaluation of PDQuest 2-D Analysis software is based on quantitative tests at three different levels of standard 2-DE analysis: spot detection, gel matching, and spot quantitation. Spot volumes were determined, corrected for background, and normalized to total spot per gel to avoid experimental variations among 2-D gels. To verify the auto detected results, all spots were manually inspected and edited as necessary. Vol % of each spot was used for quantification and *p* < 0.05 was used as criterion to define the significant difference when using paired Student’s *t*-test.

### 4.4. Trypsin Digestion and Protein Identification by MS/MS

Protein spots were carefully excised from 2-DE gels. Protein digestion with trypsin was performed according to the method described by Sghaier-Hammami et al. [30]. Spots were automatically excised, transferred to multiwall 96 plates and digested with modified porcine trypsin (sequencing grade; Promega, Madison, WI, USA) by using ProGest (Genomic solutions, Ann Arbor, MI, USA) digestion station. Gels were destained by incubation with 200 mM ammonium bicarbonate in 40% acrylonitrile (ACN) at 37 °C, twice for 30 min; then they were subjected to three consecutive dehydratation/rehydratation cycles with pure ACN and 25 mM ammonium bicarbonate in 40% ACN, and finally dried at room temperature for 10 min. 20 µL trypsin in 25 mM ammonium bicarbonate was added to the dry gel pieces and the digestion proceeded at 37 °C overnight. After finishing digestion, the peptides generated in this process were extracted with 1% trifluoroacetic acid. Tryptic peptides were analyzed by matrix-assisted laser desorption ionization time of flight mass spectrometry (MALDI-TOF/TOF MS, Applied Biosystems, Foster City, CA, USA). The matrix 4-hydroxy-α-cyanocinnamic acid was used for analyzing complex mixtures of peptides in a 4700 proteomics analyzer MALDI-TOF/TOF (Applied Biosystems, Foster City, CA, USA). For MS analysis, 800–4000 *m/z* mass range was used, 20 kV acceleration voltage and 120 ns delay extraction per spectrum. Trypsin autolysis fragments *m/z* = 842.51 and mz = 2211.10 were used to improve the mass calibration of the MALDI-TOF spectrometer. Peak lists were compared against Viridiplantae (Green Plants) or viruses data of NCBInr (National Center for Biotechnology Information) using MASCOT (http://www.matrixscience.com). Searches were performed using Cys carbamidomethylation as the fixed modification, and oxidation of methionine as variable modification, allowing 100 ppm mass tolerance in MS and 0.5 Da for MS/MS data.

### 4.5. Functional Classification of Proteins

For close identification of proteins the following criteria were used: the protein score at least 80, the coverage of protein sequence by matching peptides at least 7 or more, and at least 10% peptide sequence matches above the identity threshold. The functional information for the identified proteins was extracted from NCBI and UniProt databases. In the majority of the cases, these data were combined with literature reports, and then proteins were classified into different categories based upon their biochemical functions.

### 4.6. Statistical Analysis

One-way analysis of variance was used to identify proteins changing in abundance among those proteins present, and Tukey’s multiple range tests to detect significant differences among means of the plant treatment groups using Minitab v.8 statistical software (Minitab, State College, PA, USA). Only the spots that were present in all three replicates for at least one condition were included in the data set and the spot proteins with a test *p*-value < 0.05 were considered to show a significant change between the different experimental conditions. A list of proteins resulting as up- and downregulated was made. 

### 4.7. Design and Synthesis of Conformationally Restricted Peptides

**Figure 5:**
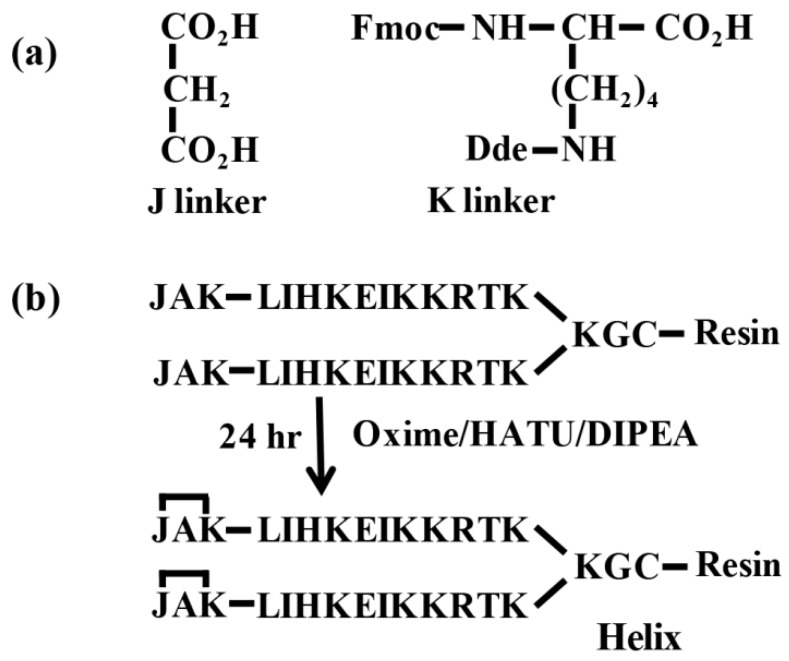
(**a**) Structure of J and K linkers. (**b**) Insertion of nucleation site into a helix-like peptide during solid-phase synthesis.

Once the identification of differentially expressed proteins was completed by proteomic strategy, we wanted to know if it was possible to identify these low concentrated proteins in leaf extracts by low-cost strategies such as immunoenzymatic methods. Exposed α-helical fragments of four proteins exhibiting significant changes in abundance after stress by rice hoja blanca virus infection were chosen for this approach. The approach used for helix-like peptide synthesis was based on replacing a structure-defining main-chain hydrogen bond (NH∙∙∙O=C-) with a covalent link (N-CO-); this was inserted into the sequence during solid-phase peptide synthesis. The covalent approach makes use of two linkers (Figure 5a) identified as J (propanedioic acid) and K (Fmoc-Lys(Dde)-OH). K linker was coupled at the end of the target sequence followed by an alanine (A) and capped with the J linker. The complete JAK cyclized capping sequence was our ‘nucleation site’ (Figure 5b). The 1-(4,4-dimethyl-2,6-dioxocyclohex-1-ylidene)ethyl (Dde) protecting group of lysine was removed with 2% hydrazine monohydrate in *N,N*-dimethylformamide (DMF) at room temperature for 3 min. The treatment with hydrazine was repeated two more times, and then the resin was washed three times with DMF. Covalent link formation and the cycling used to obtain the nucleation site occurred because of the reaction between carboxyl group (J) and amine group (K). The peptide dendrimers were synthesized on a Fmoc-MBHA Rink resin (loading: with 0.12 mequiv/g) following the Fmoc/tBu (fluorenylmethyloxycarbonyl/tert-Butyl) strategy [31]. Peptide dendrimers (10 mg/mL) were desalted with Sephadex G-10 (Amersham Pharmacia, Piscataway, NJ, USA) and oxidized with 10% dimethyl sulfoxide (DMSO) aqueous solution at pH 7.0, for obtaining the tetrameric molecules [12].

### 4.8. Characterization of Products

Peptide dendrimers were analyzed by RP-HPLC using a XBridge^TM^ BHE130 C-18 4.6 × 100 mm column (Waters Corp., Milford, MA, USA) with a 0–70% (*v*/*v*) acetonitrile linear gradient for 8 min, measured at 214 nm. Molecular mass was confirmed by a Microflex MALDI-TOF mass spectrometer (Bruker Daltonics, Billerica, MA, USA), using the α-cyano-4-hydroxycinnamic acid matrix (CHCA). Secondary structure of peptide dendrimers was analyzed in a circular dichroism (CD) spectrometer Jasco J-815 (Jasco Corp., Tokyo, Japan). Peptide dendrimers were dissolved in water (1 mg/mL) and then an aliquot of 35 µL was taken and mixed with 215 µL of 2,2,2-trifluoroethanol (30% aqueous solution). The CD spectra were obtained at 20 °C in the wavelength range from 190 to 250 nm.

### 4.9. Inoculation of Mice with Peptide Dendrimers

Polyclonal antibodies were generated against peptide dendrimers in seven week-old female CF-1 mice. Three mice per group were immunized subcutaneously at 1, 14, and 28 days with 100 µg of each peptide, diluted 1:1 in FIS (FISEAIIHVLHSR) as a T helper cell activator and 100 μL of Freund’s adjuvant (Thermo Scientific, Waltham, MA, USA). At day 42, the mice were anesthetized and bled. Two mL of blood were collected from each mouse, centrifuged at 1000 g for 5 min and the serum stored at −20 °C. The reactivity of the sera was evaluated against each peptide dendrimer by ELISA assays. This study was carried out in accordance with Law 20380 regarding animal welfare, as set out by the Chilean Health Ministry in the use of wild or protected animal species in biomedical research and approved by the Pontificia Universidad Católica de Valparaíso Bioethical Committee (ethic approval number: BIOEPUCV-A159-2014.). 

### 4.10. ELISA Assays

To determine the reactivity of anti-dendrimer antibodies maxisorp 96-well microassay plates (Nunc Thermo Scientific, Schwerte, Germany) were coated with 100 µg/mL of 10 µg/mL peptide dendrimers in coating buffer (0.01 M PBS pH 7.2). Plates were incubated overnight at 4 °C, and excess coating solution was removed by three washing cycles with washing buffer (0.01 M PBS pH 7.2, 0.05% Tween 20). Each well was then blocked using a volume of 200 μL/well of 5% non-fat dry milk/0.1% Tween 20 in 0.01 M PBS pH 7.2 and incubated for 1 h at 37 °C. The mice antisera (anti-peptide dendrimers) were diluted in serial dilutions from 1:200 in blocking buffer and 100 μL added to the wells. The plates were incubated for 1 h at 37 °C followed by five washing cycles. Goat anti-mouse IgG (H+L) HRP (32430 Invitrogen) 1:7000 was used as secondary antibody. Five washing cycles were applied after incubating the plates for 1 h. Finally, the plates were incubated with tetramethylbenzidine substrate (solution A, TMB; solution B, H_2_O_2_, 1:1 *v*/*v*, from Kirkegaard & Perry). The enzymatic reaction was stopped with H_3_PO_3_ after 15 min. Absorbance was measured at 450 nm using ELISA plate reader.

The same protocol was used to determine some of these low-concentrated proteins in leaf extracts, with the difference being that maxisorp 96-well microassay plates were coated with 100 µL of leaf extract (22 µg/mL of total protein) in coating buffer (0.01 M PBS pH 7.2). 

## 5. Conclusions

RHBV produced chlorotic spots as well as streaks in the oldest leaves 30 days after inoculation. These results indicate that an alteration in the chloroplast metabolism is produced in response to RHBV infection. Consequently, the virus-infected plants showed significant differences in morphology and metabolism, compared to normal plants. Moreover, the occurrence of RHBV altered protein expression of multiple pathways may result in: (i) changes in the composition and secretion of cell wall structural polymers; (ii) synthesis and release of defensive molecules, such as benzoxazinoids and phytolexins, to improve the defensive capability of plants against virus; (iii) synthesis of various defense related proteins including antioxidant enzymes to eliminate overaccumulated ROS; (iv) changes in expression of G-proteins orphytohormone-mediated signal transduction pathways to affect the expression of defense-related genes.

The oxidative stress is more pronounced during the development of the disease (30 days post-infection) judging from the increase in oxidative stress parameters as well as the imbalance in the antioxidative systems, mainly at the chloroplast level. Proteomic analyses showed that most of the changes produced by RHBV infection with regard to protein expression at the cellular level were related mainly to photosynthesis and carbohydrate metabolism. It seems that RHBV infection has some direct or indirect effect on PSII, by decreasing the amount of Rubisco, oxygen evolving enhancer, and PSII stability factor proteins. The results indicate that the symptoms observed in rice leaves could be due to an imbalance in antioxidant systems as well as to an increased generation or reactive oxygen species in chloroplasts, probably induced by a disturbance of the electron transport chain, suggesting that chloroplasts can be a source of oxidative stress during disease development. 

Our results suggest that the response of plants to RHBV infection can activate some defense mechanisms which include metabolic pathways related to the antioxidant system, photosynthesis, and carbohydrate metabolism. The differences observed in the proteins of the aforementioned metabolic pathways between the two varieties may contribute to clarifying the resistance observed in the variety Fedearroz 2000, and could provide a basis for further studies on new varieties.

Frequently, α-helix regions of proteins are important recognition motifs for protein–protein and protein–nucleic acid interactions that include signal transduction, transcription, apoptosis, and immune responses. However, the removal of these recognition motifs from the ordered tertiary structure of proteins results in flexible peptides that exhibit reduced target affinity and low proteolytic stability. Peptide dendrimers with the JAK nucleation site produced circular dichroism spectra typical of α-helix while peptide dendrimers without the JAK nucleation site did not. Sera from mice inoculated with these peptide dendrimers were able to specifically recognize the corresponding proteins in leaf extracts from rice plants. This approach showed how conformationally restricted peptide dendrimers to α-helix configuration can be successfully synthesized to be used for biological applications.

## Figures and Tables

**Table 1:** MS/MS identification of differentially expressed proteins under non-infected insect and virus-infected insect treatments, in both Fedearroz 2000 (Fed.) and Colombia I (Col. I) varieties (relative abundance). Plants were cultivated in a growth chamber under 12/12 h light/dark cycle at a temperature of 25 °C, and relative humidity of 77%. The plants were irrigated daily with water for approximately 30 days to keep the soil moist. The unstressed and stressed plants were kept in the same growth chamber. CI% = protein score; proteins with 55 or more were considered successfully identified. Treatment Insect = non-infected insect, Treatment Virus = virus-infected insect. Trend: increase ↑/decrease ↓ respect to control group. Proteins used in the serological assay are highlighted in grey.

Protein Name	NCBI Accession No.	CI%	Peptide Count	Experimental MW (Da)	Experimental pI (pH)	Treatment Insect/Trend (up/down)		Treatment Virus/Trend (up/down)	
						Fed.	Col. I	Fed	Col. I
**Photosynthesis**									
ATP synthase CF1 alpha subunit	NP_039380.1	100	26	92,670	6.32		↑	↓	
ATP synthase CF1 beta subunit	YP_052756.1	100	21	91,890	5.08		↑	↑	↑
Chloroplast 23 kDA polypeptide of photosystem II	ABY52939.1	100	13	22,310	5.99		↓	↓	↓
Ribulose bisphosphate carboxylase/oxygenase activase	ABG22613.1	100	22	59,230	5.23		↑	↓	
Photosystem II stability/assembly factor HCF136	Q5Z5A8.1	100	19	57,750	6.02	↓			↑
Oxygen evolving complex protein 1	2002393A	100	16	40,020	5.0	↓	↓	↓	↓
Photosystem I reaction center subunit IV	XP_015647314.1	100	8	17,380	5.87	↓	↓	↓	↓
RuBisCO	AGC93385.1	100	11	17,030	7.3	↓	↓	↓	↓
LHCII type I chlorophyll a/b binding protein	NP_001304221.1	100	9	28,160	5.11	↓	↑		
**Antioxidant System**									
Catalase	BAA81672.1	100	16	100,920	7.22	↑	↑		
Federroxin-NADP(H) oxidoreductase	ABR26171.1	100	18	49,180	6.13	↑	↓		↓
Manganese superoxide dismutase	AAA57131.1	100	11	25,350	6.19		↑	↓	↑
Superoxide dismutase (Cu-Zn)	BAA12745.1	100	6	11,460	5.67	↑	↓		↓
Glycolate oxidase	AAB82143.1	100	20	63,080	7.57		↓	↑	
**Metabolism**									
Fructose-1,6-bisphosphate aldolase	AAS05825.1	100	10	51,040	5.03	↑	↓		
Glutamine synthetase	AAM00242.1	100	9	70,970	5.10	↓	↑	↑	↑
Adenosine diphosphate glucose pyrophosphatase	BAD09955.1	100	3	21,972	5.68	↑	↓	↑	
Triosephosphate isomerase	AAB63603.1	100	11	27,790	5.24		↑	↓	↑
NAD dependent epimerase/dehydratase	EEF31135.1	100	22	57,000	6.49	↑	↓		↓
Os06g0157000	BAS96248.1	100	13	48,570	7.47			↓	
Defense									
Chitinase	AAR15893.1	100	12	35,420	6.17	↓	↓	↓	↓
Germin-like protein	BAD09958.1	100	4	23,520	6.05	↑	↑		
NBS-containing resistance like protein	ABV30845.1	0	1	18,940	5.41		↓		↑
**Biosynthesis**									
40S ribosomal protein S26	ABR25470.1	100	4	12,694.5	5.09	↓	↑	↓	
**Cellular signaling**									
FHA	XP_015638681.1	100	6	16,600	5.23	↑	↑		↑
Peptidyl-prolyl cis trans isomerase	ABR26096.1	100	11	17,080	7.12	↑	↑		↑

**Table 2:** MS/MS identification of differentially expressed proteins under non-infected insect and virus-infected insect treatments, only in Fedearroz 2000 variety (relative abundance). Plants were cultivated in a growth chamber under 12/12 h light/dark cycle at a temperature of 25 °C, and relative humidity of 77%. The plants were irrigated daily with water for approximately 30 days to keep the soil moist. The unstressed and stressed plants were kept in the same growth chamber. CI % = protein score; proteins with 55 or more were considered successfully identified. Treatment: I = non-infected insect, V = virus-infected insect. Trend: increase ↑/decrease ↓ respect to control group.

Protein Name	NCBI Accession No.	CI%	Peptide Count	Experimental MW (Da)	Experimental pI (pH)	Treatment/ Trend (up/down)
**Metabolism**						
Transketolase 1	BAD68864.1	100	17	1,321,840	5.37	↑I
GADPH	CAA30152.1	100	10	65,820	6.8	↑I
Chloroplast phosphoglycerate kinase	AAT07576	100	11	67,680	7.55	↑V
**Biosynthesis**						
MUB4	OAP05580.1	90,848	16	154,360	8	↓I,V

**Table 3:** MS/MS identification of differentially expressed proteins under non-infected insect and virus-infected insect treatments, only in Colombia I variety (relative abundance). Plants were cultivated in a growth chamber under 12/12 h light/dark cycle at a temperature of 25 °C, and relative humidity of 77%. The plants were irrigated daily with water for approximately 30 days to keep the soil moist. The unstressed and stressed plants were kept in the same growth chamber. CI % = protein score; proteins with 55 or more were considered successfully identified. Treatment: I = non-infected insect, V = virus-infected insect. Trend: increase ↑/decrease ↓ respect to control group. Proteins used in the serological assay are highlighted in grey.

Protein Name	NCBI Accession No.	CI%	Peptide Count	Experimental MW (Da)	Experimental pI (pH)	Treatment/Trend (up/down)
**Photosynthesis**						
50S ribosomal protein L21	NP_001147263.2	99,993	7	22,901.4	9.34	↑I
LHCII type I chlorophyll a/b binding protein	NP_001304221.1	100	6	27,740	5	↓I,V
**Metabolism**						
Rice alpha galactosidase	1UAS_A	100	15	66,100	6.25	↓I,V
Rieske iron sulphur protein	CAM57108	100	6	157,200	6.42	↓I,V
Fructose-1,6-bisphosphate aldolase	AAS05825.1	100	14	55,990	5.82	↑V
NAD dependent hydroxypyruvate reductase	AAS05825.1	100	14	66,101	6.51	↓I,V
Sedoheptulose-1,7-bisphosphate	AAO22559.1	100	20	60,907	5	↑I
**Defence**						
Guanine nucleotide binding protein beta subunit	AAT85192.1	100	16	52,210	6.42	↑V
Disease resistance protein (TIR-NBS-LRR class)	AEE82980.1	100	17	15,140	5.51	↑V
**Biosynthesis**						
Hypothetical protein	BAS93826.1	100	14	51,960	6.3	↑V
**Cellular signaling and transport**						
Chaperonin 21	BAD36628.1	100	8	26,780	5	↑I,V
Armadillo/beta-catenin repeat family protein	ABE66259.1	100	8	26,380	6.52	↑V
Kinesin motor family protein	AEE87012.1	100	13	14,880	6.67	↑V
Tapetum-specific zinc finger protein 1	BAA19113.1	100	8	38,040	6.55	↓I
U-box domain containing protein	NP_001147288.2	100	8	28,900	5.07	↑I,V

**Table 4:** Synthesized peptides.

Peptide	Structure	Residues	Sequence
**2237**	Linear 1a	19–29	LIHKEIKKRTK
			(LIHKEIKKRTK)2KGC
**2078**	Linear 1b		(JAK-LIHKEIKKRTK)2KGC
**2052**	Helix 1		
**2236**	Linear 2	195–205	MERYTRMSNAM
**2053**	Helix 2		(JAK-MERYTRMSNAM)2KGC
**2235**	Linear 3	32–41	EDILRSMIKE
**2054**	Helix 3		(JAK-EDILRSMIKE)2KGC
**2234**	Linear 4	190–198	QALGGHKRS
**2055**	Helix 4		(JAK-QALGGHKRS)2KGC

**Table 5:** Optical densities in specific serological recognition of rice proteins in leaf extracts using sera from mice inoculated with peptide dendrimers from two common proteins 2052 (FHA protein), 2054 (Ferredoxin-NADP(H) oxidoreductase), and two identified only in Colombia I variety 2053 (α-galactosidase), 2055 (tapetum-specific zinc finger).

Rice Variety	Group	Sera Anti-2052	Sera Anti-2053	Sera Anti-2054	Sera Anti-2055
Fedearroz	Control	0.1349 (+)	(−)	0.1174 (+)	(−)
2000	Insect	0.1327 (+)	(−)	0.1202 (+)	(−)
	Virus	0.1330 (+)	(−)	(−)	(−)
Colombia	Control	0.1326 (+)	0.1320 (+)	0.1534 (+)	0.1008 (+)
I	Insect	0.1423 (+)	(−)	(−)	(−)
	Virus	0.1158 (+)	(−)	(−)	0.1023 (+)
